# The Management of Continued Fever

**Published:** 1852-02

**Authors:** 


### BUFFALO MEDICAL JOURNAL

AND

### MONTHLY REVIEW

VOL. 7. FEBRUARY, 1852. NO. 9.

### ORIGINAL COMMUNICATIONS.

### ART. I.—THE MANAGEMENT OF CONTINUED FEVER.

By the Editor.

So many difficulties stand in the way of determining, by means of numerical analyses, the precise relative value of different measures in the treatment of diseases, that it is questionable if, by this method of study, our knowledge of therapeutics will be much advanced. The efficacy of different remedial agencies is to be estimated, *first*, by the appreciable results which follow directly their employment, and, *second*, by an apparent influence, either immediate, or more or less remote, on the severity, duration, and termination of diseases. It is, obviously, in the latter point of view, only, that statistical results can be, to any considerable extent, available. Is the ratio of mortality affected by this or that treatment? Is the severity, or the duration, of the disease greater, or less, when such and such measures are employed? These are the questions to be proposed in the prosecution of this branch of analytical investigation. The point of departure for these inquiries, it would seem, should be a knowledge of the severity, duration, and ratio of mortality pertaining to diseases uninfluenced by remedial measures. What would be the results, in these respects, of the analysis of a series of cases which were permitted to run their course without any interference from medical art? Such results would, doubtless, prove valuable contributions to medical science; but our opportunities for obtaining them, for reasons sufficiently apparent, are limited. We can only consult the interests of medical science, when the means for that end do not conflict with those humane objects of our art, which are paramount in importance. And, since we are not justified in withholding therapeutical measures which we have good grounds for thinking may exert more or less efficiency, we are, in a great measure, debarred from studying the laws of disease as respects the points mentioned, prior to instituting comparisons of results developed by the analyses of cases treated after different methods. This is one difficulty. But, were it surmountable, another, and even greater difficulty springs from the fact, that the same diseases exhibit striking diversities, at different times and places, in severity, duration, and mortality, irrespective of measures of treatment. This is not less true of Continued Fever than of any other disease. If, therefore, we had the advantage of the results of the analysis of a large collection of cases in which the disease was allowed to run its course without therapeutical interference, the laws deduced therefrom could not be taken as a fixed standard by which to measure, by comparison, the relative advantages or disadvantages of different plans of management. And, hence, it follows, that severity, duration, and fatality are not reliable as criteria for testing the remedial character of therapeutical measures. The disease may be severe, prolonged, and unusually fatal, under the most judicious management, and it may be mild, of a short career, in a large proportion of cases ending in recovery, when the treatment in nowise contributed thereto.

Were both these difficulties overcome, others would arise. The management of a disease is more or less complex. It comprises various elements. To isolate these elements severally, and assign to each its proper relative importance, is difficult and often impracticable. Statistical comparisons, to be complete, must embrace analyses of successive collections of cases that have been appropriated, respectively, to each important element which enters into the management, whether medicinal or hygienic. But the same objections are applicable to the collection of observations of this kind, as to the withholding of all remedies in order to witness the progress of disease uninfluenced by art. Aside from the intrinsic difficulties of the undertaking, moral considerations conflict with it.

Finally, assuming that difficulties did not exist, and numerical analyses were brought to bear, successfully, upon this branch of investigation, the utmost that could be attained would be to demonstrate that, in the treatment of a disease, a certain method, or plan, is preferable in the larger proportion of cases. Therapeutical principles, in other words, would be based on mean results, or averages. Now, it does not follow that measures which are most appropriate and useful in a series of cases considered collectively, are the most appropriate and useful in all the individual cases of the series. A method of treatment may be the best, for example, in seventy-five of one hundred cases, and by no means the best in the remaining twenty-five per cent. Nay, it is only a seeming paradox to assert that a method of treatment, which, if pursued indiscriminately, would be instrumental in saving more lives than any other method pursued in like manner, might yet, in some instances, directly conduce to a fatal result.

I would not be understood as denying that numerical results are of importance in the study of diseases with reference to their management. Notwithstanding the difficulties that have been mentioned, enumerations and comparisons with a view to determine the value of remedial agencies, are by no means wholly impracticable, nor altogether fruitless. Statistics showing the rate of mortality, for example, under different methods of treatment, are to be considered in estimating the relative merits of these methods. They have a certain positive value; and when this is not the case, they may possess importance in a negative sense. To illustrate the force of the latter remark, suppose, in a series of cases of Continued Fever, in which the patients were kept on gum-water for diet, the ratio of mortality was found to be not greater than in other collections in which more nutritious aliment was considered to form an important element of the management, it would certainly be a fair presumption that the latter element might be omitted with safety as respects the issue of the disease. To attempt to define the limitations of the application of the numerical method of study to therapeutics, would be to enter on a wide field of discussion entirely foreign to my present design. The foregoing remarks are merely preliminary to saying that, in writing a brief essay on the *management of Continued Fever*, I shall not enunciate conclusions based, exclusively, or mainly, on numerical observations. Aside from other considerations, the number of recorded histories that I have collected is, by far too few to furnish data for comparing the results of different methods of treatment. Nor was this an object of inquiry while the cases were in progress. The treatment pursued in all cases was that which accorded, at the time, with the opinions and judgment of the writer, without any reference to future analyses.

What are the general principles that should govern the management of Continued Fever, which are most consistent with our present knowledge of the disease, and the results of experience? In expressing views suggested by this inquiry, I shall refer to facts contained in my recorded observations in so far as they appear to have any important bearing on the subject, without descending to tedious details.

The general principles of management are applicable alike to the *Typhus*, and *Typhoid* forms of Continued Fever. It will suffice to notice incidentally any considerations pertaining specially to either type, without treating of the subject in its relations to the two types under distinct heads.

The management of Continued Fever may be considered to embrace, *first*, abortive methods, or measures designed to arrest the progress of the disease, or abridge its duration; *second*, the treatment of the disease irrespective of abortive measures, and without reference to co-existing or secondary affections; *third*, the remedies required by its complications.

### MEASURES DESIGNED TO ARREST THE PROGRESS OF THE DISEASE, OR ABRIDGE ITS DURATION.

It has long been a mooted question, whether Continued Fever, when fully formed, can be broken up by any plan of medical treatment. Various measures for that end have, from time to time, been proposed, which, after a short trial, have ceased to be employed. This fact suffices to show that no reliable means for effecting the object have, as yet, been discovered; for, if any of the measures proposed were uniformly, or in a large proportion of cases, successful, their efficacy would hardly fail to prevent their falling into disuse. The question must still be considered an open one. An opinion on the subject is, of course, but conjectural. Until our ability to control the course of the disease is demonstrated, we can only judge as to the probability or possibility of the attainment, by analogy and speculative reasoning. Both favor the expectation that a specific controlling remedy may, at some future period, be discovered. Such a remedy has been found in the case of Intermittent and Remittent Fever, by means of which, these forms of febrile disease are rendered amenable to art. Nor, admitting that all essential Fevers involve, as their primary fundamental pathological element humoral changes due to the action of special poisons, is it too much to hope that the time may come when these changes shall have been successfully investigated, their character and relations to the poisonous agents well understood, and science arrive deductively at the knowledge of opposing or counteracting remedies. Bloodletting, purging with calomel and other cathartics, emetics, vapor baths, and cold affusions are among the measures that, in past time, have had, to a greater or less extent, a transient celebrity in arresting or abridging Continued Fever. At first view, it would seem that it must be an easy task to determine, positively, respecting the efficiency of any plan of treatment instituted for these ends. It is, however, not so easy, but that measures may acquire and retain, for a time, a fictitious importance. This arises from the difficulty of demonstrating whether the febrile career has been arrested or not, owing to the fact that the symptoms of the access, not unfrequently, are observed without being followed by the formation and career of fever; and, again, we meet with instances in which febrile movement is developed, and, continuing for two or three days, ceases from its own limitations, constituting what is known as *febricula* or *ephemeral fever*. Now, we are never able to say, positively, when remedies appear to prevent the development of fever, or arrest its progress, that the same results would not have occurred spontaneously, had no remedies been employed. This being the case, it is easy to conceive that, with the influence of enthusiasm, and the bias of preconception, the efficacy of a novel method of treatment may, at first, appear to be supported by evidence which longer and more rigorous experience disproves.

Proposed methods of treating Continued Fever, with a view to rendering it abortive, of a more recent date than those which have been mentioned, are not wanting. The most notable that have fallen under my notice are, 1st, the administration of quinia in large doses. This has been advocated particularly by Thomas D. Mitchell, formerly connected with the Transylvania University, Lexington, Ky. 2d, opium given in large doses is affirmed to be, in a large proportion of cases, successful, by Dr. A. G. Henry, of Pekin, Ill., and others. 3d, the wet sheet, as employed by the hydropathists, with a view to fomentation and diaphoresis. I have made these methods the subjects of a few observations, which are by no means adequate as tests of their respective merits, and are, perhaps, hardly worth reporting. Few as they are, however, I will give a brief account of them, as a small contribution toward a collection of data which shall suffice to settle the importance to be attached to each of these methods. Such a collection, so far as I know, is not, as yet, the property of science.

In *two* cases, in the first collection, quinia was prescribed with a view to an abortive effect. In both cases, the fever was fully formed before the remedy was given. In *one* case, *twenty-four* grains of quinia were prescribed in twenty-four hours, on the first day, and twenty grains on each of the two ucceeding days. It occasioned characteristic buzzing in the ears, but exerted no appreciable effect on the progress of the disease. The duration of the fever in this case, was eighteen days. In the other case, the dose given was three grains every six hours, which was continued for six or seven days. No appreciable effect was observed in this case, the duration being fifteen days. Both cases were of the *Typhoid* type.

In another case, on the first day of admission into hospital, five grains of quinia were given, and repeated twice, under the impression that the disease was Remittent Fever. No appreciable effect followed in this case.

I have repeatedly prescribed quinia in doses of two grains every four or six hours from the time patients came under treatment, and continued the use of the remedy in this way for several days, without observing any apparent influence on the disease.

In *five* cases, of which I have presented notes, *opium* or *morphia* was given in pretty large doses, in order to try the effect of this as an abortive remedy. The histories of these cases, in so far as concerns the point under consideration, are briefly as follows:

*Case* 1. Martin Lyons, Irish, laborer, aged 33, admitted into hospital Dec. 13, 1849, on the *sixth* day after taking to the bed. The record of symptoms on the day of his admission was not made. *Four grains of opium were given at night.* At the morning visit on the day following, he was sleeping profoundly and heavily. Respiration labored, and inspiration somewhat spasmodic. He was readily roused, and replied to questions promptly and coherently. Reported feeling finely, and said he had passed a very comfortable night. Perspired copiously during the night, and slept soundly, without muttering. Surface, at time of examination, covered with a cold, clammy perspiration. Pulse 130, extremely small and feeble. Singultus.

15th. Reports comfortable. Respiration during night, labored, and inspiration somewhat spasmodic. Had epistaxis last night. Skin warm and mellow. Pulse 108, more developed. Rhythm of respiration now normal. Has cough and rusty expectoration, with physical signs of pneumonitis affecting right lung inferiorly. Skin and conjunctiva present moderate yellowness.

16th. Manifested delirium during night by incoherent talking. Appears now rational. Reports quite comfortable. Cough and expectoration continue. Epistaxis yesterday twice. Respirations 28; rhythm normal. Yellowness of skin and conjunctiva diminished. Pulse 88, tolerably developed.

17th. Symptoms not materially changed. Pulse 84.

19th. Symptoms continue unchanged. No delirium. Pulse 96.

22d. Reports "finely." Cough somewhat troublesome. No febrile movement. Appetite improving.

Parotitis commenced on the 30th, and proceeded to suppuration. On the

19th of January, he was able to sit up. Discharge from parotid much diminished. Appetite good. This is the date of the last record of the case.

*Remarks.* The remedy was given, in this case after the fever had continued for several days. It was followed by cerebral symptoms of a grave character, as denoted by the respiration, great acceleration and feebleness of the pulse, and copious perspiration. On the next day but one after the opium was administered, the course of the fever appeared to be suspended. He was laboring under pneumonitis, which, perhaps, was developed prior to the administration of the opium. This complication, and parotitis, which subsequently occurred, retarded the convalescence. There seems to be some ground for suspecting that the remedy exerted an influence over the duration of the febrile career in this case.

*Case* 2. Katharine Riley, Irish, domestic, aged 17, admitted Dec. 18, 1849, on the *third* day after taking to the bed. On the 19th, she presented a dull aspect. Appeared drowsy. Face congested; eyes slightly suffused; skin hot and dry. Moderate tympanites, and tenderness in iliac regions. Pulse 120. Slight mental aberration at night. *Four grains of opium were administered.*

20th. Reports not so well. Says she passed a restless night. Complains of pain "all over." Face deeply congested. Aspect more dull. Somnolent, but easily roused. Pulse 116. Skin dry and warm. Abdominal tenderness continues.

The career of the fever persisted without any material change, and three days after the above date, parotitis, on both sides, commenced, and proceeded to suppuration.

Jan. 9th, she was able to sit up, and rapidly convalesced.

*Remarks.* The result of the remedy, in this instance, appeared to be negative.

*Case 3d.* Michael Donnehea, aged 7, admitted P. M., Dec. 21, 1849, on the *third* day after taking to the bed.

22d. Complains of cephalalgia and debility, anorexia, thirst. Slight congestion of the face and upper extremities. Tongue thickly coated and inclined to dryness. Two or three dejections during the night. Pulse 112, small and feeble. Abdomen full, but not resonant. Moderate tenderness in right iliac region. A sparse eruption over abdomen and chest, with typhoid characters. Has had no medicine since his admission, or previously. *Opium in dose of a grain and a half was prescribed.*

23d. Reports quite well. Perspired profusely last evening. Tongue clean. No febrile movement. No dejection. No medicine prescribed.

Jan. 3d, it is noted that the patient began to sit up after date of the last record, and appeared convalescent. Day before yesterday, 1 P. M., Jan. 1st, he took to the bed, and last night exhibited delirium by incoherent talking. He complains of headache. Tongue moist and furred. Bowels moved last night. Vomited last night. Ate porridge and toast for breakfast this morning. Skin perspiring. Pulse 108. No medicine, but restrict the patient to farinacious diet.

No record of the case is made after this date. The slight relapse was possibly occasioned by over indulgence in eating.

*Remarks.* Unless the administration of the opium was a coincidence with the spontaneous cessation of the febrile career, it would seem to have been successful in arresting, at once, the progress of the disease. It will be observed that the grain and a half of opium constituted the sole medicinal treatment of the case.

*Case* 4. Thomas Toole, Irish, boatman, aged 25, admitted P. M., Oct. 14th, 1850, having been ill *five* days, but it is not noted how long he had kept his bed. 15th, he presented the following symptoms:—Considerable congestive redness of face and upper extremities; cephalalgia; thirst; some cough. Skin hot. Pulse accelerated.

16th. Cephalalgia. Congestive redness continues. Conjunctiva injected. Skin warm and dry. No dejection for three days. Pulse 104. Respirations 24. Slight tympanites.

*Sulphate of Morphia, gr. ss., at morning, to be repeated at night.*

17th. Reports better. Says he slept well. Was talkative during night. No pain in head, or elsewhere. Aspect improved. Conjunctiva injected. Congestive redness of surface continues. Skin warm and moist. Pulse 104, tolerably developed. No cough.

*Sulphate of Morphia, gr.* 1-4, *every four hours.*

18th. Manifested considerable delirium during night, by incoherent talking, and attempting to get out of bed. Pulse 116. Respirations 20, etc.

The febrile career persisted in this case; pneumonitis became developed, and death occurred on the fourteenth day after admission.

*Remarks.* Some amelioration in the symptoms was apparent on the day following that on which the grain of morphia was given, but the progress of the disease was unaffected by the remedy.

*Case* 5. Nora Kinne, Irish, aged 24, domestic, admitted on the evening of Nov. 5, 1850. Had been ill since the 1st, and had kept the bed since the 3d.

On the 6th, she presented the following symptoms:—Conjunctiva slightly injected. Moderate congestive redness of the face, and slight on upper extremities. Occasional cough. Respirations 25. Skin hot and dry. Pulse 116, moderately developed. Tongue dry, reddened and coated. Moderate meteorism. Slight tenderness in right iliac region. *Opium, grs. iv, given in the forenoon.*

At 5, P. M., sleeping soundly. Skin warm and moist. Pulse 112. Respirations 20.

7th. Reports better. Says she slept well during night. Face and hands slightly congested. Skin warm and moist. Tongue moist and thinly coated, reddened at top. Respirations 20. Pulse 78. Tympanites lessened. No dejection. No tenderness. Anorexia, thirst. *Morphia, gr. ss., at bed time.*

7th. Reports better. Says she slept well. No dejection. Urinates freely. Face and hands slightly congested. Skin warm and mellow. Tongue moist and coated. Slight tympanites. Slight tenderness in right iliac region. No eruption. Pulse 88. Respirations 20.

9th. No material change in symptoms.

10th. Several dejections during night. Skin hot and dry. Tongue dry and reddened. Pulse 92. Anorexia; thirst. Respirations 20. No tympanites. Slight tenderness in right iliac region.

11th. Reports still better. Says she slept well. Skin warm and mellow. Tongue moist and furred. Pulse 84. Anorexia. No thirst. Sat up a little.

12th. Symptoms denote improvement. Some appetite.

13th. Improving.

She continued to improve until the 18th, when relapse of fever commenced. On the 19th, the pulse was 108 at A. M., and at P. M., 120. On the 20th, it was 128 at A. M., and at P. M., 144. On the 21st, the symptoms exhibited decided improvement, the pulse having fallen to 90. From that date, she again convalesced, and left hospital on the 2d December.

*Remarks.* There is room for the conjecture that the morphia exerted an influence on the progress of the fever in this case. The striking improvement in the symptoms on the day succeeding that on which the morphia was given, favors this supposition. This improvement was especially manifested in the pulse. Convalescence could be pronounced in three or four days afterward. Relapse of fever occurred in several of the cases observed through the winter of 1850–51, as will be perceived on reference to the second report.

The facts contained in the foregoing cases do not, of course, authorize any positive conclusions. So far as they go, however, they furnish encouragement for the supposition that opium, in large doses, in some cases, does possess more or less power to affect the progress of Continued Fever. A series of recorded cases, showing the results of this plan of treatment, is certainly to be desired. It is to be remarked that, in each of the foregoing cases, the career of the fever had continued for several days before the treatment was pursued. It is, perhaps, reasonable to suppose that the chances for the success of any method of abortive treatment would be greater the earlier it is resorted to. Nor was the opiate plan of treatment carried to the full extent recommended by Dr. Henry and others. The minimum dose proposed with a view to abortive effects was employed; and the plan was not carried out by repeating the measure once, twice, or more, in the same case.

I have resorted to the wet sheet in *five* instances, of which I will give a succinct account.

*Case* 1. An account of this case I must give from recollection. The patient was a private inmate of the hospital, in the summer of 1850, out of the period of my service, and the details of the history were not noted. He presented sufficient evidences of Continued Fever, having been ill several days, but had not taken to the bed prior to his admission. On the morning of the day following that on which he was admitted, the wet sheet was applied, and the body then wrapped in blankets, after the plan of *packing* practised by the hydropathists. These were allowed to remain for four hours. Copious diaphoresis followed. The skin became cool, and he passed a comfortable night. During the previous night he had exhibited delirium, and at the morning examination, before the wet sheet was applied, there existed high febrile movement, with somnolency, etc. On the next day, he was free from fever; skin cool, pulse not accelerated, mind perfectly clear, and, in short, the febrile career seemed at once and completely arrested. I was delighted with the apparent success of the measure. In the afternoon I received a message that the patient thought to be doing so well in the morning was dying. I found him in apoplectic coma, and death occurred the succeeding night. I ascertained that, feeling so well in the forenoon, he had essayed to sit up; and had actually dressed himself, and sat up for a time. He had returned to bed, and fallen into a state of unconsciousness before an unfavorable change in the symptoms attracted attention. The apoplectic coma and fatal termination, it is not improbable, were due to the imprudent act just stated. Although they could not, with propriety, be charged upon the remedy, the unfortunate issue of the case deterred me for some time from again resorting to it, notwithstanding the immediate apparent effect was striking and satisfactory.

*Case* 2. James Cavernaugh, aged 15, Irish, admitted Dec. 2d, 1850, having been attacked the previous day, with pain in head and limbs, chills, anorexia, thirst, etc. The symptoms, on his admission, were as follows:—Complains of debility. Lies quietly in bed. Aspect bright. Mind clear. Anorexia. Tongue moist and thinly coated. Not much thirst. Vomited to-day. Tenderness over right iliac region and epigastrium. No tympanites. Pulse accelerated. Skin hot and dry. *Pulv. Doveri, gr. iv, every four hours.*

3d. Complains of severe pain in head. Says he did not sleep during night. Face and hands considerably congested. Conjunctiva slightly injected. Skin hot and dry. Pulse 112, well developed. Anorexia. Tongue moist and coated. Considerable thirst. No dejection. Tenderness over epigastrium and right iliac region. *S. morphia, gr.* 1-4, *at bed time.*

4th. Reports feeling very badly. Complains of pain in head. Says he did not sleep during night. Aspect dull. Face and upper extremities present considerable congestive redness. Skin hot and dry. Pulse 108. Tongue moist and coated. Anorexia. Thirst. Vomited last evening. Slight tenderness over abdomen generally. Moderate meteorism. No dejection since the commencement of illness. Says he has no cough. Slight epistaxis this morning.

*The wet sheet was applied for two hours*, after which, morphia, gr. 1—4, and brandy, ℥ss., were administered.

At evening, he reported better than at morning. Pain in head continues, but is lessened. Skin warm and moist. Tongue coated, dry in the center, and moist at sides. Anorexia. Not much thirst. No nausea. Moderate meteorism continues. One dejection. Pulse 116.

5th. Reports better. Aspect improved. Skin warm and dry. Tongue coated and inclined to dryness. Anorexia. Some thirsty. No nausea. Some tenderness in epigastrium and right iliac region. Slight meteorism. No eruption. No dejection. Slight vomiting. Pulse 104.

6th. Reports much better. Aspect much improved. Says he slept well. Face much less congested. Skin warm and mellow. Pulse 84. Tongue moist and coated. Anorexia. No thirst. No nausea.

7th. Symptoms improving. Pulse 76.

8th. Improving. Tongue moist and clean, except at base. Some appetite. Pulse 84.

Convalescence continued until the 18th, when relapse of fever occurred, and continued for three or four days, when he again convalesced without any untoward symptoms.

*Remarks.* Distinct amelioration of the symptoms followed the application of the wet sheet, which was resorted to on the *third* day, and convalescence could be pronounced on the *eighth* day after taking to the bed. It may be reasonably surmised that the remedy affected the progress of the disease in this instance.

*Case* 3. Patrick Cavernaugh, aged 9, brother of James, (case 2,) admitted Nov. 27th, 1850. He complained of debility at time of admission, but was dressed, and kept about until Dec. 2d, when he took to the bed. On the 5th of December, he presented the following symptoms:—Cephalalgia. Face flushed, and upper extremities slightly congested. Skin hot and dry. Pulse 116. Tongue dry and coated. Anorexia. Considerable thirst. No dejection for two or three days. Moderate meteorism. Tenderness in right iliac region. No eruption. Slight cough. *The wet sheet was applied at 11 A. M., and continued two hours.*

7 P. M. Reports better. Less pain in head. Face flushed, and perspiring. Skin warm and moist. Tongue moist and coated. No thirst. Moderate meteorism. No dejection. Pulse 124.

A small dose of morphia was administered after the wet sheet was removed, and brandy ℥ss given twice within four hours.

5th. Reports better. Was talkative during night, and frequently got out of bed. Replies now to questions promptly and rationally. Aspect improved. Face slightly flushed. Skin warm and mellow. Tongue moist and thinly coated. Has some appetite. No thirst. Slight meteorism. No dejection. Vomited slightly this A. M. Pulse 88. Slight cough. No eruption.

6th. Reports free from pain, but very weak. Attempted, frequently, to get out of bed during early part of night.

Directions had been given to administer morphia according to circumstances, and, by inadvertency, a quarter of a grain was repeated four times (i. e. one grain,) within the space of three hours. At the morning examination, he appeared partially narcotized, being roused with some difficulty. He slept profoundly the latter part of night. Face somewhat congested, and upper extremities slightly. Skin warm and mellow. Tongue dry and thinly coated. No appetite. No thirst. Occasional nausea. No dejection for five or six days. Moderate meteorism. Slight abdominal tenderness. No eruption. Pulse 96.

6th. Reports comfortable. Says he slept well. Slight congestion of face and upper extremities. Skin warm and dry. Tongue rather dry and thinly coated. Some appetite. Some thirst. One dejection. No meteorism. Pulse 84. Disposed to somnolency.

8th. Aspect much improved. Tongue moist and nearly clean. One dejection, *moulded, and perfectly healthy in appearance.* Pulse 96. Skin warm and mellow. No delirium.

This patient convalesced until the 15th, when relapse of fever occurred, the duration of which is not recorded; but it continued a few days only.

*Remarks.* The history of this case shows a striking amelioration of the symptoms after the application of the wet sheet, but not an immediate arrest of the progress of the disease. The large quantity of morphia given on the second night after the wet sheet was applied, is a point of interest. It is difficult, of course, to say what influence these measures exerted, singly or combined, upon the duration of the disease; but it is a fair presumption, that the short career may have been due to one or both of them.

*Case* 4. Margaret O'Connor, aged 14, Irish, domestic, admitted March 12th, 1851. She had kept the bed for two days prior to her admission. The day but one after her entrance, i. e. March 14, she presented the following symptoms:—Cephalalgia. Cheeks flushed. Anorexia. Thirst. Tongue thinly coated, and dry in center. No nausea nor vomiting. Slight cough. Pulse 104. Skin warm and dry. No eruption. Moderate meteorism. No dejection. No tenderness.

*Wet sheet was applied.*

15th. Reports much better. Aspect much improved. The record states as follows:—"She had the wet sheet at 7 P. M. Prior to its being applied, the skin was hot and dry. She said she was comfortable during its application. The surface became cool and moist, and so continued. She slept well. Thirst ceased. Skin is now cool and moist. Pulse 72. Tongue moist and thinly coated. Says she has some appetite, and has taken some toast and tea with relish. Respiration normal. No cough."

It was directed to repeat the wet sheet if febrile movement returned. It was not called for. She convalesced from the date of the preceding record, no medicines being administered until the 17th, when quinia, grs. ii, three times daily, was directed. No relapse of fever occurred in this case.

*Remarks.* With the *post hoc* reasoning, the remedy, in this instance, was directly and completely successful.

*Case* 5. This was a case of fever occurring, in private practice, in a child four years of age. The case is interesting from the intensity of the febrile movement, and from the fact that the wet sheet constituted the chief treatment.

The patient came under my care March 10th, A. M. The day previous, she fell asleep after dinner, and on awakening, complained of feeling very ill, and took to the bed. She had not appeared well for several days previously, but, on the morning of the day of the attack, was as bright as usual. During the night after the attack, she vomited several times; the surface was hot; she was very restless, and talked incoherently. On the morning of the 10th, just before I saw her, she had a slight convulsive tremor, without entire loss of consciousness, lasting but for a moment or two. The pulse was greatly accelerated, and the skin very hot.

I directed simply the ext. Hyos., in solution, grs. ii, every four hours.

At noon, high febrile movement continued; skin intensely hot; pulse ranging from 170 to 180; disposed to somnolency; incoherency. The bowels had moved the day previous. Under the impression that the disease would prove to be scarlatina, the chlorinated soda in solution was prescribed, but occasioning vomiting, it was omitted after the second dose.

At evening, the symptoms presenting no change, *the wet sheet was applied.* The immediate effect was soothing, and the pulse fell to 160. Late at night, the rhythm of the respiration became somewhat affected, the inspiration being shortened and quickened, and the expiration prolonged. Dr. Winne visited in consultation; and it was agreed to envelop the body in a sheet wetted with mustard water, and, afterward, to repeat the wet sheet as before, ice being applied to the head. The sheet wetted with mustard water was applied for an hour, and followed by the simple wet sheet, which was continued for about four hours. The patient was soothed, but the pulse remained the same. No remedies were given, except the hyosciamus.

11th. At 6 A. M., patient had slept quietly for several hours. Respirations 40, rhythm normal. Pulse 160. Less delirium. Asked frequently for cold water.

The ext. Hyos. was continued, and the face, neck and arms frequently sponged with cool water. At noon, a solution of sulphate of morphia and tartrate of antimony and potash, of each gr. 1-32, every three hours, was substituted for the hyosciamus. No vomiting since the chlorinated soda was suspended. No dejection until an enema was administered in the afternoon, which was followed by a tolerably free evacuation. Abdomen not meteorized nor tender. Pulse 160.

12th. Passed a comfortable night. Slept quietly much of the time. Pulse, at A. M., 150. Skin less hot, mellow. No vomiting. No dejection. Slight meteorism. No food had been given since the attack prior to this morning, when she took a little milk and essence of beef. She relished the latter, and it was continued through the day. She had a little wine and wine whey. At evening, she took a little bread and milk which she had asked for. Pulse, at evening, 140. Skin mellow. Respirations 30. No dejection. Slight epistaxis. Slight mental aberration.

The treatment, on this day, consisted of the morphia and antimony, of each gr. 1-32, every four hours.

14th. Night before last was passed quite comfortably. Yesterday morning, aspect improved. Took a little egg-nog and a little bread and milk with relish. Pulse ranged from 130 to 140. Bowels moved slightly by enema. No medicine until evening, when the ext. hyos. gr. i ss, every four hours, was prescribed. To-day, aspect still more improved. Pulse 130, and at noon, 120. Syrup of rheubarb and magnesia was prescribed in repeated doses, until it produced a laxative operation.

From this date the little patient rapidly convalesced without farther treatment. On the 17th, medical attendance was discontinued, and she has remained perfectly well up to the present time, Jan. 1852.

It will be understood that the foregoing report of cases in which quinia, opium, and the wet sheet were employed, contains the sum of my observations, up to this time, relating to these methods. The cases are not selected for illustration, but are all that I have collected bearing on the present subject. As already remarked, they are too few for purposes of induction. So far as they go, however, they afford evidence, in the first place, of the safety of making trial of these methods. This is a point of primary importance. With the present uncertainty attending the employment of any measures to cut short, or abridge, the duration of Continued Fever, we should be hardly justified in resorting to those which, if not successful, would be likely to impair the chances of passing through the disease with safety. In this respect the three methods of treatment just mentioned compare favorably with others that have formerly been in repute, viz: copious bloodletting, emetics, and cathartics.

But the facts contained in the histories of the cases in which opium and the wet sheet were employed, although insufficient to authorize inductions respecting the positive influences due to them, yet tend to support the presumption that they do possess more or less power to ameliorate the symptoms, and to affect both the duration and severity of the disease. At all events, they are of a character to render it interesting to prosecute farther observations, in order to collect a larger series of data for investigation.

It is by no means improbable that various methods of treatment may be found to exert more or less control over the intensity and persistence of the morbid conditions upon which Continued Fever depends. This is the fact with respect to periodical fevers. In the latter form of fever, arsenic, strychnia, prussiate of iron, salacine, etc. are found to have each a specific influence, the quinia being superior to any. So, in Continued Fever, it should be, not only an object of inquiry, to ascertain a single plan of abortive treatment which may be, to a greater or less extent, successful, but to discover different methods; and, finally, by comparisons, to ascertain their relative value, and the circumstances upon which the efficiency of each depends. Should Science, at length, succeed in acquiring a special remedy, adequate to the objects of art in the management of this disease, as quinia is in the treatment of periodical fevers, it will certainly be not more extraordinary than that the latter should have been discovered; nor, prior to this discovery, were there stronger reasons for anticipating it than now exist with reference to prospective success in seeking for the means of controlling Continued Fever.

A single remark suggests itself, in conclusion, respecting measures that have heretofore been considered successful in arresting or abridging the career of Continued Fever. Allusion has been made to the factitious importance attached, for a transient period, to different methods of treatment for these ends. It is to be considered, on the other hand, that the demonstration of this fact with regard to any measure naturally leads to a disparagement of the value which it may actually possess. If a remedy fails to fulfill the sanguine expectations which had been raised, it becomes as much undervalued as it had been over estimated, and, hence, falls into unmerited neglect. Possibly this remark may be applicable to some of the methods of treatment supposed to be successful in controlling the course of Continued Fever. The extent however to which some of these methods have been employed forbids such a supposition. For examples, bloodletting, emetics, and cathartics, it is probable, have been in vogue sufficiently to establish their inefficacy as abortive remedies. It may, perhaps, be doubted whether this is true of cold affusions as recommended by Dr. Currie, about a half century ago. The striking success which appeared to attend this measure, and which gave it, for a short time, such celebrity, it would seem, could hardly have rested on a basis wholly supposititious. May not disappointment at not finding it so uniformly or completely successful as had been anticipated, have caused its value to be unduly depreciated, and too soon abandoned? Having no facts to contribute bearing on this question, I shall content myself with merely propounding it.

### TREATMENT IRRESPECTIVE OF ABORTIVE MEASURES, AND WITHOUT REFERENCE TO CO-EXISTING OR SECONDARY AFFECTIONS.

What are the leading principles that should govern the management of Continued Fever, exclusive of measures designed to arrest its career or abridge its duration, and aside from remedies addressed to local affections, with which the disease may become complicated? Several considerations may be mentioned which have an important bearing on this inquiry.

*First.* The disease, if not arrested, or abridged, by therapeutical interference, has its intrinsic limitations. It runs a certain course, ending in recovery, after the lapse of one, two, or three weeks, provided the issue be not fatal.

*Second.* The intimate pathological conditions upon which the disease is dependent, or, in other words, its proximate cause, in the present state of science, cannot be explained. The nature of the special agents inducing the disease is unknown, and, consequently, their mode of action within the organism, as well as the immediate changes produced thereby, are not understood. We have not, therefore, the point of departure from which to advance deductively toward a rational plan of cure.

*Third.* In the majority of instances, the tendency of the disease is to end favorably. If we study the histories of a series of fatal cases, we find that the unfavorable issue is generally owing to causes not belonging intrinsically to the disease — for examples, various complications like pneumonitis; or what may be distinguished as accidents, like hæmorrhage, intestinal perforation, apoplectic coma; or external circumstances, such as the absence of hygienic advantages; or diminished power of resistance from enfeebled health, etc., etc. All these are not, properly, elements of the disease, some being incidental to it, and others wholly adventitious. Divested of every thing collateral, or superadded, affecting its progress in an unfavorable manner, there is reason to believe that the termination in recovery might almost be considered to be a law of the disease.

Now, from these considerations, it appears to be a legitimate deduction that the management of Continued Fever *per se*, under the qualifications embraced in this division of the subject, does not claim efficient medicinal interference. This conclusion accords with the views entertained by many of the most judicious practitioners and the soundest writers of the present time. Apart from means instituted to cut short the disease, and to prevent or relieve complications, the plan of treatment most consistent with what knowledge we at present possess, is the *expectant*, as it is called. The mere fact of the existence of Continued Fever does not involve any special therapeutical indications. Cases may occur in which little or no active interference is called for. It is probable that, of a collection of cases permitted to run their course, under favorable hygienic conditions, without medicinal treatment, a good proportion would end in recovery. This remark is not based altogether on conjecture. For example, Hildebrand says of Typhus: "The experience of all ages proves that, like all other contagious fevers, it is frequently cured of itself, that is, by the mere action of the vital powers, unassisted by any of the resources of our art. The simple state of the disease is, in fact, always dissipated in this manner; and it would be a lamentable circumstance, especially for the poor, if this assertion were not correct." The same author, after citing his own case in which, after an emetic and bleeding, he took nothing but lemonade and barley water during the whole course of the disease, continues: "I have repeatedly seen patients who were affected with a simple ordinary typhus, get perfectly well by taking nothing but lemonade." He adds, "For this mode of treatment I am indebted to the views of a great physician, the Baron De Stork, who has treated the same disease successfully by the simple employment of wine whey." In several of the cases which I have observed, the medicinal treatment has been virtually nugatory, consisting only of slightly palliative remedies, or placebos. As an instance, in one of the cases contained in the first collection, the patient, who had been under homœopathic management for the first ten days, and then abandoned by the attending practitioner as a hopeless case, my treatment, aside from diet, and other measures of hygiene, consisted solely of the sulphate of quinia, gr. 1, every four hours, the career of the disease running to twenty-eight days, and then ending favorably. This was a case in private practice, and the chief reason for prescribing the small doses of quinia was, that I might not in the event of an unfavorable issue, be exposed to the charge of having given no remedies!

That Continued Fever may often do well without medicinal interference, is a fact important to be borne in mind; but it does by no means follow that the rule of management of the disease is to be based thereon. In saying that the treatment should be *expectant*, it is not intended to assert that the practitioner is to be merely a spectator of the phenomena of the disease, waiting for convalescence without any effort to render therapeutical aid.

The point to be enforced in view of the considerations that have been presented is, that the presence of the disease does not, as a matter of course, furnish the occasion for active remedies. We are not to resort to efficient medication simply because a patient is affected with Continued Fever, nor are perturbatory or powerful measures always required. On the other hand, it may be that little or no medicinal treatment is called for. The therapeutical indications, in other words, are not indissolubly associated with the existence of fever, but are derived from the various circumstances, incidental and accidental, which may be connected with the disease in individual cases. To watch, in every direction, for the development of these indications, to appreciate their character and importance, and to meet them, as they arise, by appropriate measures, are, in general terms, the objects of the *expectant* method of management.

Let us now proceed to inquire respecting some of the particular indications for treatment during the progress of Continued Fever. They may be divided into *special* and *general.* The *special* indications will relate, for the most part, to the symptoms and events, respectively, which enter into the natural history of the disease. The most convenient arrangement will be to notice them under the same anatomical divisions employed in the analyses upon which the preceding Reports were based. Following this plan, the indications pertaining to the *nervous system* are first in order. The symptoms which claim attention with reference to treatment under this head are, *cephalalgia, somnolency, insomnia,* and *delirium.*

The pain in the head which accompanies the access and early part of the febrile career, is sometimes acute, and may be the chief source of suffering. It is to be borne in mind that, in a greater or less degree, it is an element of the disease, and that, after a few days, it usually ceases to be the subject of complaint. It does not necessarily denote inflammation, or even a tendency thereto, although it may be intense. A different opinion was held by practitioners heretofore, and even now obtains to a considerable extent. In connection with delirium it has been supposed to denote encephalitis, or a liability to this complication, which, in fact, occurs as rarely as it was formerly regarded frequent. The treatment, then, which this symptom may call for, is not to relieve or avert inflammation, as was laid down, for example, in the treatise by Dr. Southward Smith, an able work that did much toward giving direction to the practical notions of physicians in this country, which were formed twenty years ago. The author just named advocated prompt and copious bloodletting for that end. It is questionable how far the cephalalgia is due to active congestion, or any disorder of the circulation. The propriety of bloodletting, theoretically, is involved in this question. The power of this remedy to affect pain or other cerebral symptoms must be mainly by diminishing the quantity of blood determined to the brain, directly, by the amount abstracted from the mass, or, indirectly, by lessening the force and rapidity of the circulation. The circumstances which indicate the remedy, therefore, are those showing the dependence of the symptoms which we wish to relieve upon repletion of the vessels of the encephalon, or upon conditions of the vascular system which depletion will be likely to remove. The subject of bloodletting, in general, in Continued Fever, will be more properly noticed presently, in another connection. Of the efficacy of this remedy in relieving intense cephalalgia, I cannot speak from results witnessed in cases the histories of which I have preserved. I have resorted to it for this or any other purpose in two only of the one hundred analyzed; but in a large proportion of the cases collected, pain in the head had ceased to be a prominent symptom before the patients came under my care.* I have the same confession to make respecting topical depletion by cupping or leeches. Evaporating lotions applied to the forehead, and over the whole head, afford comfort; and in order to facilitate their application, as well as to promote, in itself, coolness of the surface, *cutting the hair close to the scalp*, whenever circumstances render it proper, is a measure of some consequence. The latter is a standing direction for all fever patients admitted into hospital during my term of service. The *ice cap*, in some cases in which the cephalalgia was intense, I have found useful. Cold affusions, limited to the head, pouring water for several minutes from some height from a pitcher or jug, the head being held over a basin, is an appropriate means of procuring the benefits of refrigeration. I cannot speak of its advantages from trial, never having tried it. It is said to be strikingly efficient, and with regard to its immediate effects an error of observation would not be very likely to occur. An *opiate*, in tolerably large doses, for example a quarter of a grain of morphia, has seemed to me to be serviceable in relieving this symptom, when not intense, and unaccompanied by evidences of marked determination of blood to the head. I have never observed any unpleasant effects to follow the use of this remedy in the early period of febrile career when cephalalgia is present.

This symptom by no means gives rise to a special indication for treatment in all cases. If moderate or slight in degree, with the prospect that it will gradually abate as the disease progresses, and in a few days cease, minor palliative remedies will be sufficient.

*Somnolency*, in other words a semi-conscious condition from which the patient is readily, but only momentarily roused, need not deter us from prescribing opiate or anodyne remedies, but, on the contrary, they appear to produce a good effect by substituting for this condition more complete and refreshing sleep. The results of my analyses show that the state of somnolency, which is somewhat characteristic of Continued Fever, more especially the *Typhus* type, does not denote a tendency to stupor or coma which would contra indicate narcotic medicines. The latter symptoms, which denote great

---

* In a few of the cases bloodletting had been practiced before the patients came under my observation. This will account for an apparent discrepancy between this statement and some facts mentioned in the preceding Reports.

danger, are of infrequent occurrence, and belong properly among the events complicating the disease.

*Vigilance*, or insomnia, is a symptom, claiming attention in the management. Is it not probable that the enfeebled condition of the brain inducing the delirium and mental hebetude incident to the progress of Continued Fever may be due, in some measure, to the deficiency of sleep? It is to be borne in mind that *somnolency* is not sleep, and, hence, that patients may be in a dozing state the greater part of the time, and still experience the evils of insomnia. To endeavor to procure sleep is an important indication, and for this end opiates seem to me to be valuable remedies throughout the disease. Opium, in substance or tincture, Dover's powder, or morphia may be prescribed. I have been accustomed to employ the last article mentioned oftener than the others. In a large proportion of the cases upon which my second Report was based, generally in doses of a quarter of a grain every four or six hours, it was given during the greater part of the febrile career. In several cases this constituted the chief, and in some instances almost the sole treatment. It has never occasioned any appreciable ill effects; and with reference to this and other indications, I am disposed to think that an anodyne influence maintained by some form of opiate is of considerable utility in the management of Continued Fever.

*Delirium*, if slight, or even moderate, does not furnish special therapeutical indications. It is an element of the disease, and, if it does not exceed the degree in which it is present in the larger proportion of cases, is not of much importance. When it is prominent as a symptom, and especially when it is predominant, or active, it, of course, claims attention. Opiates, in decided doses will be found useful. I have in several instances observed, after complete sleep effected by anodyne remedies, the mind become more clear, and if delirium recurred, it was lessened in degree. But, so far as my experience goes, the remedy upon which most reliance is to be placed for restraining the delirium of Continued Fever, is the *Tartrate of Antimony and Potassa*, given in connection with anodynes. It is seldom that this remedy will fail to palliate this symptom, and generally the effect is striking. Small doses usually suffice. My course is, to direct a *sixteenth* or *eighth* of a grain to be given hourly, or half hourly, until the patient becomes quiet, unless distinct nausea or vomiting occurs, when it is to be suspended. The desired quietude will frequently be obtained without its being carried to an extent to disturb the stomach. Anodynes may at the same time be given with intervals of four or six hours as when the object is to procure sleep. The manifestations of delirium calling for treatment take place especially at night, and, hence, the tartar emetic is seldom required during the day time, but it may be repeated nightly if required.

So satisfactory has been the employment of antimony with reference to this symptom, that, excepting in those instances in which the delirium was remarkably active and persistent, a more efficient remedy seems to me hardly attainable, or even desirable. In the instances in which delirium is a predominant feature, I have no remedies to suggest more likely to be beneficial than opiates and antimony. There is reason to think that when the disease is thus characterized, it will almost inevitably prove fatal in spite of any measures of treatment.

The indications pertaining to the *Digestive System* will relate, mainly, to *nausea and vomiting, diarrhœa, hæmorrhage from the bowels, constipation, tympanites, and abdominal tenderness.*

*Nausea and vomiting*, seldom present in a degree to require special treatment, very rarely occur after the access, or early part of the febrile career. When they claim any attention, restrictions as respects the quantity and quality of what is ingested will usually suffice; and, if further measures are necessary, those are appropriate which are employed to relieve gastric irritation developed irrespective of fever, or in connection with other affections. These measures are a sinapism or blister to epigastrum; morphia; kreosote; hydrocyanic acid, etc.

*Diarrhœa*, is a symptom embraced among the distinctive traits of the *Typhoid* type, which will sometimes call for special remedies. So far as my experience goes, it is seldom difficult to regulate the evacuations by simple measures. An anodyne and astringent enema, repeated according to circumstances, usually suffices; and if morphia, or some form of opiate, is administered with reference to other objects, agreeably to the plan I have pursued in the majority of cases, it is seldom that the diarrhœa will require additional treatment. The presence of diarrhœa furnishes an indication, in addition to those already noticed, for the employment of some form of opiate. I have had no experience with the nitrate of silver which has been recommended to be given by the mouth, and by injection, to relieve the diarrhœa occurring in *Typhoid* fever. The spirits of turpentine also, which has been advised for the same end, I have prescribed too little to form an opinion of its efficacy from the results of my own observations. Merely looseness of the evacuations, without undue frequency, does not call for special treatment.

In the two instances among the one hundred cases upon which the preceding Reports were based, characterized by the occurrence of *hæmorrhage from the bowels*, the administration, by the mouth, of opium and the acetate of lead in one case, and, by enema, of the tincture of opium and tannic acid in the other case, proved at once successful. This symptom, which is somewhat distinctive of the *Typhoid* type is probably due to ulceration of Peyer's patches, conjoined with a condition of the blood itself, favoring its transudation. The importance of pursuing measures to arrest the hæmorrhage as speedily as possible, cannot be doubted. This is apparent from the prostration attending its occurrence. Having once occurred in the progress of a case, the impropriety of administering cathartics, or laxatives subsequently, is obvious.

*Constipation* not infrequently exists in Continued Fever, oftener in the *Typhus*, than in the *Typhoid* type, but occasionally in the latter; that is to say, the bowels will remain quiescent for several successive days if cathartics or laxatives are not given, and, especially if opiates enter into the management.

The question here suggests itself, what are the indications in the management of the disease which pertain to cathartics? Most writers on this subject enjoin the employment of remedies of this class sufficiently to procure one or more dejections daily; and the majority of practitioners, probably, make this an object of treatment. My own observations lead me to hold a different view. The results of the investigation of the histories that I have collected show, *first*, that diarrhœa frequently follows the operation of a cathartic; *second*, that the proportion of cases characterized by diarrhœa is greatest among those in which cathartics were employed; *third*, in one case, hæmorrhage from the bowels followed the operation of doses of castor oil, occurring only in this connection, and no cathartics being given in that case with these exceptions; and, *fourth*, no appreciable evils resulted from omitting all remedies of this class in a large proportion of the cases, a dejection not occurring, in several instances, for periods varying from three to eight days.

These facts appear, to my mind, to render more than doubtful the propriety of resorting to cathartics in Continued Fever, save for some special objects.

Looking at the subject rationally, it is to be considered, *first*, that cathartics are not indicated, more than other remedies, by the mere fact of fever being present. We have no adequate evidence that patients pass through the disease more safely and pleasantly, other things being equal, when cathartics enter into the management, than when they do not. *Second*, it is a principle of general application in therapeutics, not to be lost sight of here, more than in other affections, that remedies of more or less potency, if not indicated, will be likely to prove hurtful. If not efficient for good, they will probably be productive of harm in proportion to their activity. They cannot be expected to be neutral in their consequences. *Third*, it is not difficult to conceive, from the *modus operandi* of cathartic remedies, how they may prove injurious. Their local action on the alimentary canal involves more or less irritation, and this, when the follicular patches are diseased, ulcerated, in the *Typhoid* type, (a condition not incompatible with constipation,) cannot but be prejudicial. Under these circumstances the only way in which they can be considered appropriate in their local effects is to suppose that by effecting the discharge of the morbid contents of the intestinal canal, they remove causes of irritation, which, by remaining, would do greater harm than is occasioned by the action of the cathartic. Admitting that the direct effects of the latter are bad, it may be said that it is indirectly useful by causing the discharge of what would occasion effects much worse. This is doubtless the reasoning often adopted; but the premises are assumed, not proved. Indeed, in so far as the facts presented in the preceding Reports furnish evidence relating to the matter, it is adverse to the hypothesis just stated. Other reasons which may be assigned such as, 'to correct the secretions,' 'to excite the action of the liver,' etc., are too gratuitously speculative to deserve attention, more particularly so long as the results of experience appear to be adverse to the utility of cathartics.

The remote, or general effects of this class of remedies is to diminish the vital forces, to conduce to prostration. In other words they are debilitating in their tendency, and, hence, it is reasonable to suppose that they may be productive of harm rather than good. Although this phraseology is somewhat indefinite, owing to the want of precision in our knowledge of the remote effects of cathartics, as well as various other remedies, on the organism, it conveys ideas which are practically appreciable, and with which all practitioners are sufficiently familiar. For the reasons, therefore, which I have thus endeavored briefly to set forth, I think purgatives are not useful in the management of Continued Fever except there arise some special indications for their use. Constipation alone may furnish an indication. If the bowels do not move spontaneously after several days, even if no evils are apparent, it may be the part of prudence to effect a movement. But for this, so far as my experience goes, active cathartics are not requisite. Remedies distinguished as *laxatives* will suffice, and even these, simple injections will usually render unnecessary. The latter are always to be preferred when practicable or adequate. If remedies are given by the mouth, the mildest, and most grateful are preferable, such as Seidlitz powders, Congress-water, Magnesia, etc., provided there are no special reasons for moving the bowels except to obviate costiveness.

By omitting cathartic or laxative measures, unless they appear to be specially called for, the evacuations which take place spontaneously will sometimes preserve an appearance of health, a fact which perhaps will hardly seem credible to those who are accustomed to move the bowels by medicinal means daily, or every other day throughout the progress of the disease. This fact is noted in the histories of several of the cases which I have observed.

*Tympanites* or *meteorism*, may furnish an indication for cathartics. This symptom is a source of distress, and, by impeding respiration, etc., may add to the danger of a fatal issue. It therefore claims attention. Enemas are sometimes useful; but if these are ineffectual, a cathartic should be tried, more especially if the bowels are constipated. Mechanical compression by means of a bandage, and friction with stimulating embrocations appear to be serviceable. Cloths dipped in heated spirits of turpentine have seemed to be a useful application. I have given, in several instances, powdered charcoal with a view to its property of absorbing gases, and I have imagined that it produced some effect in that way.

*Abdominal tenderness*, is peculiar to *Typhoid* cases, and is seldom so great as to occasion suffering except upon pressure, or movements of the body. Fomentations, or a light cataplasm, would be appropriate, but the cases must be rare in which this symptom requires special treatment, excluding, for the present, those in which peritonitis exists as a complication.

It is rarely that important indications appertain to symptoms referable to the *Pulmonary System*, excluding, for the present, cases in which the disease becomes complicated with pneumonitis. More or less cough belongs to the natural history of Continued Fever, but it is seldom sufficiently prominent, or troublesome, to need remedies. When it is otherwise, provided opiates do not enter into the management, small doses of morphia dissolved in mucilage, or syrup, are appropriate.

*Epistaxis*, which also belongs to the natural history of the disease, in the great majority of instances is slight, not requiring to be restrained, but perhaps being a salutary fluxion. Occasionally, however, it is copious, and may proceed to an alarming degree. The usual methods of arresting hæmorrhage from the nostrils, are then to be resorted to, viz: the application of cold, and, in some cases, plugging the anterior and posterior nares. Of the one hundred cases upon which the preceding Reports were based, in but one case was the loss of blood so great as to occasion apprehensions of danger. The hæmorrhage was arrested in this case without resorting to plugging.

The indications pertaining to the *circulation* relate chiefly to *bloodletting*. Respecting bloodletting in Continued Fever, my recorded observations are, with a very few exceptions, confined to cases in the treatment of which it did not enter. That I have omitted to avail myself of facilities to study, to some extent, the effects of this therapeutical measure, has been not wholly owing to a reluctance to employ a remedy of doubtful applicability, but, in some measure, to the fact that a large proportion of the cases, the histories of which I have preserved, did not come under observation until the disease had advanced so far that bloodletting would have been deemed inadmissible by those who advocate its propriety.

If Continued Fever offers indications for bloodletting, it is evident that they are now of a character different from those which have heretofore led to its employment in this disease. The objects for which this measure was formerly practiced, (aside from the effort to cut short the disease,) were to relieve and to prevent local inflammations. Its supposed utility was based either on the dogma that fever was always symptomatic of local inflammation somewhere, after the doctrine of Broussais, or on the belief that inflammations of internal organs were very liable to occur as complications, and that, in the latter cases, they constitute the chief source of danger. The doctrine held by Dr. Southward Smith, for example, was, that the only element of fever over which art can exert any control, is the tendency to local inflammations, and hence, bleeding was regarded by him as (to use a common metaphor) the sheet anchor of the treatment. These pathological notions are now well nigh obsolete. Few will be found, at the present time, to contend that there is no such disease as essential fever; and it may be considered equally settled that local inflammatory complications, calling for active treatment, are of infrequent occurrence. These facts by no means suffice to disprove the utility of bloodletting, but they show, assuming its usefulness, that the reasons why it is so, are other than those which have heretofore been considered satisfactory.

In seeking to form a just estimate of the value of this, as of every other remedy, the question of course arises, what are the accumulated results of experience? This question properly precedes an inquiry concerning the rational indications which the remedy is to fulfill. The difficulties, already alluded to, of testing by experimental observations, different methods of treatment, are applicable to bloodletting, as well as other therapeutical measures. It cannot be said that the positive influence exerted by the employment of this remedy on the average fatality, or duration of Continued Fever has been settled by numerical facts. It may, indeed, be doubted if statistics have demonstrated, or are capable of demonstrating that it possesses any power to shorten the career of the disease, or to lessen the liability to death, all other things being equal. It must, however, be admitted that experience renders highly probable, if it has not established an important negative conclusion, viz, that bloodletting does by no means always increase the duration, or fatality of Continued Fever. This is a legitimate deduction from the employment of the remedy in successive series of cases, in which the average of fatality and duration is small or moderate. Not to mention other statistics bearing on this point, those contained in the treatise by Dr. Southward Smith, before referred to, suffice for this deduction. These statistics are based on cases, in a large proportion of which bloodletting was practiced, it being regarded as the most important therapeutical measure in the management of the disease, and the results, during several years, do not exhibit a large proportion of deaths.

Now, while a low rate of mortality, on the one hand, and, on the other, a high rate, may not afford much evidence either for, or against the effect of bloodletting, owing to the great variations of the disease at different times and places, in the relative fatality, and for other reasons, a small proportion of deaths in a series of cases into the treatment of which the measure entered, certainly affords a strong presumption that the effect was not in a great degree prejudicial.

Were we to inquire concerning the views which have been entertained by practical men, and professedly based on impressions derived from individual experience, we should find discrepancy of opinion. It would require considerable research to determine on which side recorded testimony of this kind preponderates. Unanimity of opinion by no means obtains among practitioners of the present time. Confidence in the remedy and its employment have, doubtless, declined within the few past years, but the subject is an open one for discussion and investigation. Common consent, or the united voice of the profession, does not fix a precise estimate of the value of the remedy, more than do the rigorous analyses of facts.

With our present knowledge, it would appear to be as unphilosophical and irrational to commit the judgment in behalf of repudiation of bloodletting in all cases of Continued Fever, as to advocate its employment in every case. The conclusion most consistent with facts, and which best reconciles the conflicting results of experience, is, that the remedy may, or may not be appropriate — that it is useful in some cases, and probably hurtful in other cases. Its propriety and usefulness, it is reasonable to suppose, depend not only on the circumstances appertaining to individual cases, but on characters of the disease peculiar to certain times and places; and, hence, observations showing at one time, or place, its utility, and at another time or place its evils, may be equally correct. The importance of studying the traits of Continued Fever, as of various diseases, of different seasons and situations, in order to adapt the treatment to the varying characters which it may assume, applies to other therapeutical measures, as well as the particular remedy under consideration. The value of this precept, emphatically enunciated by Sydenham, the experience of practical observers since the day of that illustrious physician has abundantly confirmed.

In view of the general conclusion, with respect to bloodletting, just stated, it remains to inquire what are the indications derived from symptoms which should lead us to resort to the remedy in individual cases of Continued Fever? Let it be borne in mind that we are not to bleed simply because the patient has Continued Fever; we do not bleed for the disease *per se.* We do not bleed to extinguish the disease, or because it is proved that by this remedy its duration is shortened. Nor do we bleed because Continued Fever necessarily involves local inflammation, or tends thereto. What do we immediately effect by bloodletting? We lessen the mass of blood, and thereby diminish the motive power carrying on the circulation. Now the quantity of blood may be in morbid excess in fever, and the momentum may be unduly exaggerated. Here, then, are objects to be fulfilled by detracting blood, viz: to reduce the excess in quantity of the circulating fluid, and to abate the excess of energy in the action of the forces carrying on the circulation. If this be the correct rationale of the supposed beneficial operation of bloodletting, the special indications, it is plain, relate to the condition of the vascular system, as respects repletion, and of the circulation as respects power. Fullness of the vessels, a pulse denoting, not *activity*, but *power*, i. e. in volume well developed, and resisting compression — these symptoms, associated with the other elements of a high grade of febrile movement, are those by which the employment of this measure is to be governed.

To settle upon the conditions under which bloodletting may be practiced, is one point, but another point, manifestly of importance, is, to determine the extent to which it may be carried with prudence and advantage. The limitations of the remedy, in the abstract, are defined with more difficulty than the indications for its employment. In resorting to this, as to any potent therepeutical measure, the harm that it is capable of doing, is to be taken into account, as well as the good that it is desired to effect by it. The chief point of consideration in this aspect is, that since we do not expect to arrest or abridge the continuance of the disease, it would be obviously injudicious to bleed to an extent to compromise the ability of the patient to hold out through the febrile career. It were better to forego the relief it may afford to some of the contingent symptoms, than to incur that hazard. To adapt the remedy to the circumstances of particular cases, keeping within proper conservative bounds, is a matter that cannot be embraced in routine rules, but must be left wholly to the judgment and tact of the practitioner.

To conclude these few desultory hints on the subject of bloodletting in Continued Fever, I should say, judging from my own observations, that the indications for this remedy are rarely present. I can readily understand, however, that in a series of cases of a different description it may be otherwise. A large proportion of the cases I have observed were among newly arrived immigrants, reduced by the deprivations incident to a tedious voyage across the Atlantic, and suffering from the depressing moral influences of sickness in a hospital in a strange land. Moreover, the greater number, as already remarked, were admitted after more or less continuance of the disease, and the rational indications for bleeding are very rarely present save at an early period. Patients attacked in vigorous health, and especially if laboring under an overplus of blood, and an unduly energetic circulation, other things being equal, are those who would be expected to present indications for bloodletting. A larger proportion of this class of patients would be likely to be found in a series of cases in a healthy rural district, than among the poor population of a city. Hence, I have had occasion to remark that practitioners in the country are apt to be stronger advocates of bloodletting than those residing in cities.

Finally, the indications for bloodletting are less frequently present in the *Typhus*, than in the *Typhoid* type. This accords with the distinctive characters of the former. In the *Typhus* form of the disease the vital powers are more prostrated; the circulation seldom exhibits morbid increase of power, and the importance of conservative precautions is greater than in *Typhoid.*

